# Tumor polo-like kinase 4 protein expression reflects lymphovascular invasion, higher Federation of Gynecology and Obstetrics stage, and shortened survival in endometrial cancer patients who undergo surgical resection

**DOI:** 10.1186/s12905-024-02911-9

**Published:** 2024-02-07

**Authors:** Qinyan Zhao, Minli Wang, Mingcong Chen

**Affiliations:** Department of Radiation, Taizhou Cancer Hospital, Taizhou Key Laboratory of Minimally Invasive Interventional Therapy & Artificial Intelligence, Taizhou, 317502 China

**Keywords:** Polo-like kinase 4, Surgical endometrial cancer, Tumor characteristics, Disease-free survival, Overall survival

## Abstract

**Background:**

Polo-like kinase 4 (PLK4) serves as a marker for tumor features and poor outcomes in cancers. This study aimed to explore the associations of tumor PLK4 protein expression with tumor characteristics and survival in endometrial cancer (EC) patients who underwent surgical resection.

**Methods:**

This study included 142 EC patients who underwent surgical resection. Tumor tissue samples were obtained for tumor PLK4 protein expression detection via immunohistochemistry (IHC).

**Results:**

Among EC patients, 26.1% had a PLK4 IHC score of 0, 24.6% had a score of 1–3, 27.5% had a score of 4–6, and 21.8% had a score of 7–12. Tumor PLK4 protein expression positively associated with lymphovascular invasion (*P* = 0.008) and Federation of Gynecology and Obstetrics (FIGO) stage (*P* = 0.005). Disease-free survival (DFS) was not different between patients with tumor PLK4 IHC scores > 0 and ≤ 0 (*P* = 0.154) but was reduced in patients with scores > 3 vs. ≤ 3 (*P* = 0.009) and > 6 vs. ≤ 6 (*P* < 0.001). Similarly, overall survival (OS) was not different between patients with scores > 0 and ≤ 0 (*P* = 0.322) but was shorter in patients with scores > 3 vs. ≤ 3 (*P* = 0.011) and > 6 vs. ≤ 6 (*P* = 0.006). After adjustment, a tumor PLK4 IHC score > 6 (vs. ≤ 6) (hazard ratio (HR): 3.156, *P* = 0.008) or > 3 (vs. ≤ 3) (HR: 3.918, *P* = 0.026) was independently associated with shortened DFS and OS.

**Conclusion:**

A tumor PLK4 IHC score > 6 or > 3 associates with shortened DFS and OS in EC patients who undergo surgical resection.

**Supplementary Information:**

The online version contains supplementary material available at 10.1186/s12905-024-02911-9.

## Impact statement

What is already known on this subject?

- PLK4 is reported to serve as a marker for tumor features and poor outcomes in gynecologic malignancy. It may be a key oncogene of EC by regulating cancer stem cell characteristics, but lacking clinical evidences.

What do the results of this study add?

- This study not only explored the distribution of tumor PLK4 protein expression in EC patients who received surgical resection, but also investigated its linkage with tumor characteristics and survival using multiple cut-off value.

What are the implications of these findings for clinical practice and/or further research?

- Tumor PLK4 protein expression reflects lymphovascular invasion and higher FIGO stage in EC patients who receive surgical resection. Meanwhile, its IHC score > 6 and > 3 serves as an independent indicator for shortened DFS and OS, correspondingly, suggesting its ability for predicating disease progression and survival outcomes of these patients.

## Background

Endometrial cancer (EC) is a common gynecologic malignancy in perimenopausal and postmenopausal women, ranking sixth among female cancers in terms of incidence and mortality, with 417,367 new cases and 97,000 deaths in 2020 worldwide [1, 2]. Age, obesity, diabetes, hypertension, and unopposed estrogen are well-established risk factors for EC [2]. In the early stage, EC is often characterized by abnormal uterine bleeding, with approximately 75%-90% of EC patients presenting this symptom [3, 4]. For patients in Federation of Gynecology and Obstetrics (FIGO) stage I-III (and in some cases, FIGO stage IV), the primary treatment involves surgery, and it can be combined with adjuvant radiotherapy or chemotherapy for those at high risk [5]. However, the mortality rate of EC is high and continues to increase, reaching 2.0–3.7 per 100,000 women in 2020 [6, 7]. Therefore, it is meaningful to find potential biomarkers to facilitate the screening of EC patients with a high risk of a poor prognosis, further realizing individualized treatment and improved clinical outcomes.

Polo-like kinases (PLKs) are a family of serine/threonine kinases, including PLK1, PLK2, PLK3, PLK4, and PLK5, characterized by a conserved N-terminal kinase catalytic domain and a C-terminal noncatalytic domain containing two or three polo-boxes [8]. In contrast to PLK1, PLK2, PLK3, and PLK5, which have two distinct polo boxes, PLK4 possesses three polo boxes and consists of 970 amino acids with a molecular mass of 109 kDa [9]. All PLKs except PLK5 are involved in centrosome biology and cell progression regulation, influencing various cellular functions critical for homeostasis, development, and tissue repair [9]. Among them, PLK4 is a pivotal regulator of tumor growth and metastasis, making it a therapeutic target for cancers [10].

The role of PLK4 in gynecologic malignancy has been reported [11]. A study showed that PLK4 transcription is regulated by the human papillomavirus (HPV) E7 protein through disrupting the DREAM-CDE/CHR pathway in HPV-infected cervical cancer samples, indicating the potency of PLK4 as a biomarker for HPV-infected cervical cancer [11]. Clinically, another study indicated that PLK4 is overexpressed in epithelial ovarian cancer tissues compared with benign tissues and is related to shortened survival in these patients [12]. In addition, a bioinformatic analysis suggested that PLK4 may be a key oncogene of EC that acts by regulating cancer stem cell characteristics [13]. Based on the aforementioned evidence, PLK4 is assumed to be a potential biomarker reflecting recurrence and death risk in EC patients. However, PLK4 has not been determined in EC patients so far.

Hence, this study explored the distribution of tumor PLK4 protein expression in EC patients who underwent surgical resection as well as its association with tumor characteristics and survival using multiple cutoff values.

## Materials and methods

### Subjects

This retrospective study included 142 EC patients who underwent surgical resection between April 2017 and December 2022. The inclusion criteria were as follows: patients were 1) diagnosed with EC by pathology; 2) more than 18 years of age; 3) underwent surgical resection; 4) had available and well-preserved tumor tissue samples that could be used for immunohistochemistry (IHC) assays; 5) had complete clinicopathological data; and 6) had at least one follow-up data point for analyses. The exclusion criteria were as follows: patients with 1) other malignant tumors; 2) hematological malignancies; 3) blood coagulation dysfunction. The study protocols were submitted to the institutional review board from our hospital, and approval was obtained for study implementation. Informed consent was obtained from each subject.

### Data acquisition

The features of EC patients (age, menopausal status, comorbidities, histological subtype, invasion status, International FIGO stage, cancer antigen (CA) and carcinoembryonic antigen (CEA) abnormities, adjuvant radiotherapy, and adjuvant chemotherapy) were acquired from the electronic medical record system. CA125 ≥ 35 U/mL, CA19-9 ≥ 37 U/mL and CEA ≥ 5 ng/mL was defined as abnormality [14]. In addition, follow-up data were screened; routine follow-up was conducted until June 2023 (once every 2 months in the first year and once every 3 months thereafter). Among the follow-up data, recurrence or death statuses and the corresponding time periods were extracted. On this basis, disease-free survival (DFS) and overall survival (OS) rates were computed.

### IHC assay

Tumor tissue samples from EC patients were obtained for PLK4 detection via IHC assay. Briefly, a primary antibody (1:100, Anti-PLK4 rabbit polyclonal antibody, No. Cat. ml089343, Shanghai Enzyme-linked Biotechnology Co., Ltd., China) was added, and incubation was conducted overnight at 4 °C. Then, a secondary antibody (1:100, RBITC-conjugated goat anti-rabbit IgG, No. Cat. ml087493, Shanghai Enzyme-linked Biotechnology Co., Ltd., China) was added, and incubation was conducted for 20–30 min at room temperature. After that, colorant was added for staining. The color staining was terminated when a positive result appeared and the background was clearly observed under the microscope. Finally, the samples were scored according to the intensity and density of staining. The final IHC score ranged from 0 to 12, which was calculated by multiplying the intensity of staining score (ranging from 0 to 3) and the density of staining score (ranging from 1 to 4) [15].

### Tumor PLK4 IHC scores

To explore the association between tumor PLK4 IHC scores and the features of EC patients, patients were sorted into one of four groups based on the tumor PLK4 IHC score: IHC score = 0, 1–3, 4–6, or 7–12.

### Statistics

SPSS v.26.0 (IBM Co., Ltd., USA) was used for data analyses. The normal distribution was checked using the Kolmogorov–Smirnov test. Associations between features and four levels of IHC scores were determined by the chi-square test or Fisher’s exact test (unordered categorical features) or Kruskal‒Wallis H test (ordinal categorical features). The DFS and OS rates were assessed with the Kaplan‒Meier estimator, and comparisons of DFS/OS rates between tumor PLK4 IHC scores were conducted with the log-rank test. Univariable and forward stepwise multivariable Cox regression analyses were utilized to examine risk factors for DFS or OS. A *P* < 0.05 was considered to indicate statistical significance.

## Results

### Clinical features

In EC patients who underwent surgical resection, the mean age was 60.1 ± 9.0 years. There were 29 (20.4%) premenopausal patients and 113 (79.6%) postmenopausal patients. A total of 96 (67.6%), 15 (10.6%), 19 (13.4%), and 12 (8.5%) patients had endometrioid carcinoma G1/G2, endometrioid carcinoma G3, serous endometrial carcinoma, and clear cell endometrial carcinoma, respectively. There were 55 (38.7%) patients with myometrial invasion ≥ 50%. A total of 106 (74.6%) patients experienced no or epithelial cervical invasion, and the other 36 (25.4%) patients experienced stromal cervical invasion. In terms of FIGO stage, 88 (62.0%), 18 (12.7%), 23 (16.2%), and 13 (9.2%) patients had stage I, II, III, and IV disease, respectively. The detailed clinical features are listed in Table 1.

**Table 1:** Features of EC patients

Features	EC patients (*N* = 142)
Age (years)	
Mean±SD	60.1±9.0
≥60 years, No. (%)	73 (51.4)
Menopausal status, No. (%)	
Pre-menopause	29 (20.4)
Post-menopause	113 (79.6)
Diabetes, No. (%)	33 (23.2)
Hypertension, No. (%)	56 (39.4)
Histological subtype, No. (%)	
Endometrioid carcinoma G1/G2	96 (67.6)
Endometrioid carcinoma G3	15 (10.6)
Serous endometrial carcinoma	19 (13.4)
Clear cell endometrial carcinoma	12 (8.5)
Myometrial invasion ≥50%, No. (%)	55 (38.7)
Cervical invasion, No. (%)	
None or epithelial	106 (74.6)
Stromal	36 (25.4)
Lymphovascular invasion, No. (%)	36 (25.4)
FIGO stage, No. (%)	
I	88 (62.0)
II	18 (12.7)
III	23 (16.2)
IV	13 (9.2)
CA125 abnormity, No. (%)	45 (31.7)
CA19-9 abnormity, No. (%)	41 (28.9)
CEA abnormity, No. (%)	55 (38.7)
Adjuvant radiotherapy, No. (%)	108 (76.1)
Adjuvant chemotherapy, No. (%)	46 (32.4)

CA125 ≥35 U/mL, CA19-9 ≥37 U/mL and CEA ≥5 ng/mL was defined as abnormality

*EC* endometrial cancer, *SD* standard deviation, *FIGO* the International Federation of Gynecology and Obstetrics, *CA125* cancer antigen 125, *CA19-9* cancer antigen 19-9, *CEA* carcinoembryonic antigen

### Tumor PLK4 protein expression

**Fig. 1:**
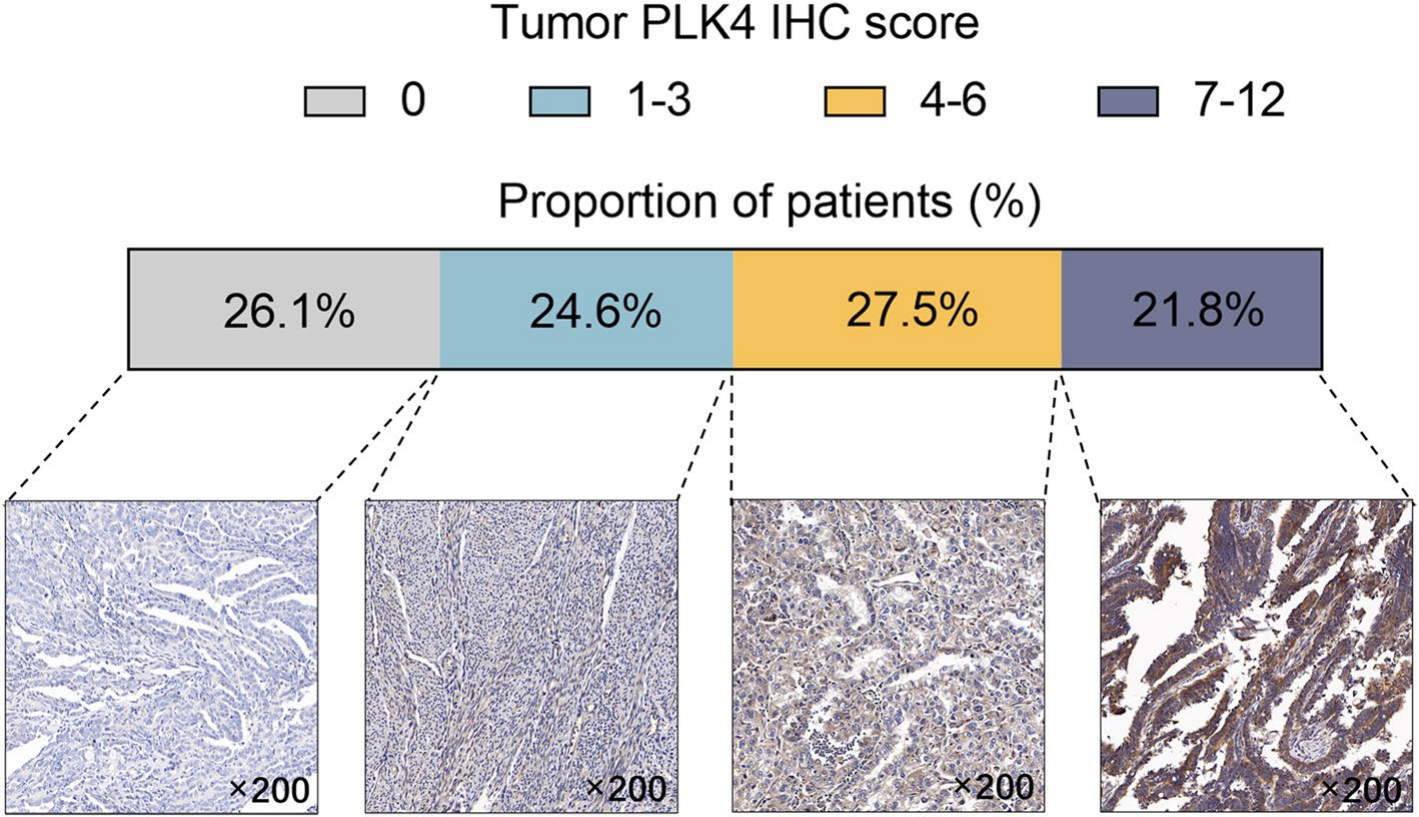
The distribution of tumor PLK4 IHC scores in EC patients who underwent surgical resection

Tumor PLK4 protein was mainly expressed in the cytoplasm or membrane. The mean tumor PLK4 IHC score was 3.8 ± 3.3, ranging from 0 to 12, in EC patients who underwent surgical resection. There were 26.1% patients with a tumor PLK4 IHC score = 0, 24.6% patients with a score of 1–3, 27.5% patients with a score of 4–6, and 21.8% patients with a score of 7–12. Examples of tumor PLK4 IHC scores = 0, 1–3, 4–6, and 7–12 are presented in Fig. 1.

### Comparison of tumor PLK4 protein expression among patients with different clinical characteristics

Tumor PLK4 protein expression was elevated in patients with lymphovascular invasion compared to those without (*P* = 0.008). Tumor PLK4 protein expression was increased in patients with higher FIGO stage than those with lower stage (*P* = 0.005). No difference was found in tumor PLK4 protein expression among patients with other different clinical characteristics, including age, menopausal status, diabetes, hypertension, histological subtype, myometrial invasion, cervical invasion, cancer antigen (CA) 125 abnormity, CA19-9 abnormity, carcinoembryonic antigen (CEA) abnormity, adjuvant radiotherapy, and adjuvant chemotherapy (all *P* > 0.050, Table 2).

**Table 2:** Correlation of tumor PLK4 IHC scores with features

Features	Tumor PLK4 IHC score				*P* value
	0 (*n*= 37)	1-3 (*n* = 35)	4-6 (*n* = 39)	7-12 (*n* = 31)	
Age, No. (%)					0.346
<60 years	14 (37.8)	20 (57.1)	21 (53.8)	14 (45.2)	
≥60 years	23 (62.2)	15 (42.9)	18 (46.2)	17 (54.8)	
Menopausal status, No. (%)					0.916
Pre-menopause	8 (21.6)	8 (22.9)	8 (20.5)	5 (16.1)	
Post-menopause	29 (78.4)	27 (77.1)	31 (79.5)	26 (83.9)	
Diabetes, No. (%)					0.055
No	26 (70.3)	24 (68.6)	36 (92.3)	23 (74.2)	
Yes	11 (29.7)	11 (31.4)	3 (7.7)	8 (25.8)	
Hypertension, No. (%)					0.815
No	20 (54.1)	22 (62.9)	25 (64.1)	19 (61.3)	
Yes	17 (45.9)	13 (37.1)	14 (35.9)	12 (38.7)	
Histological subtype, No. (%)					0.408
Endometrioid carcinoma G1/G2	23 (62.2)	27 (77.1)	29 (74.4)	17 (54.8)	
Endometrioid carcinoma G3	3 (8.1)	3 (8.6)	3 (7.7)	6 (19.4)	
Serous endometrial carcinoma	8 (21.6)	3 (8.6)	5 (12.8)	3 (9.7)	
Clear cell endometrial carcinoma	3 (8.1)	2 (5.7)	2 (5.1)	5 (16.1)	
Myometrial invasion ≥50%, No. (%)					0.130
No	28 (75.7)	20 (57.1)	24 (61.5)	15 (48.4)	
Yes	9 (24.3)	15 (42.9)	15 (38.5)	16 (51.6)	
Cervical invasion, No. (%)					0.085
None or epithelial	31 (83.8)	26 (74.3)	31 (79.5)	18 (58.1)	
Stromal	6 (16.2)	9 (25.7)	8 (20.5)	13 (41.9)	
Lymphovascular invasion, No. (%)					0.008
No	34 (91.9)	21 (60.0)	31 (79.5)	20 (64.5)	
Yes	3 (8.1)	14 (40.0)	8 (20.5)	11 (35.5)	
FIGO stage, No. (%)					0.005
I	29 (78.4)	19 (54.3)	27 (69.2)	13 (41.9)	
II	5 (13.5)	2 (5.7)	4 (10.3)	7 (22.6)	
III	3 (8.1)	10 (28.6)	5 (12.8)	5 (16.1)	
IV	0 (0.0)	4 (11.4)	3 (7.7)	6 (19.4)	
CA125 abnormity, No. (%)					0.148
No	28 (75.7)	22 (62.9)	30 (76.9)	17 (54.8)	
Yes	9 (24.3)	13 (37.1)	9 (23.1)	14 (45.2)	
CA19-9 abnormity, No. (%)					0.327
No	27 (73.0)	27 (77.1)	29 (74.4)	18 (58.1)	
Yes	10 (27.0)	8 (22.9)	10 (25.6)	13 (41.9)	
CEA abnormity, No. (%)					0.659
No	25 (67.6)	19 (54.3)	25 (64.1)	18 (58.1)	
Yes	12 (32.4)	16 (45.7)	14 (35.9)	13 (41.9)	
Adjuvant radiotherapy, No. (%)					0.298
No	11 (29.7)	11 (31.4)	6 (15.4)	6 (19.4)	
Yes	26 (70.3)	24 (68.6)	33 (84.6)	25 (80.6)	
Adjuvant chemotherapy, No. (%)					0.181
No	28 (75.7)	19 (54.3)	29 (74.4)	20 (64.5)	
Yes	9 (24.3)	16 (45.7)	10 (25.6)	11 (35.5)	

CA125 ≥35 U/mL, CA19-9 ≥37 U/mL and CEA ≥5 ng/mL was defined as abnormality

*PLK4* polo-like kinase 4, *IHC* immunohistochemistry, *FIGO* the International Federation of Gynecology and Obstetrics, *CA125* cancer antigen 125, *CA19-9* cancer antigen 19-9, *CEA* carcinoembryonic antigen

### Comparison of DFS and OS between patients with different tumor PLK4 protein expression levels

**Fig. 2:**
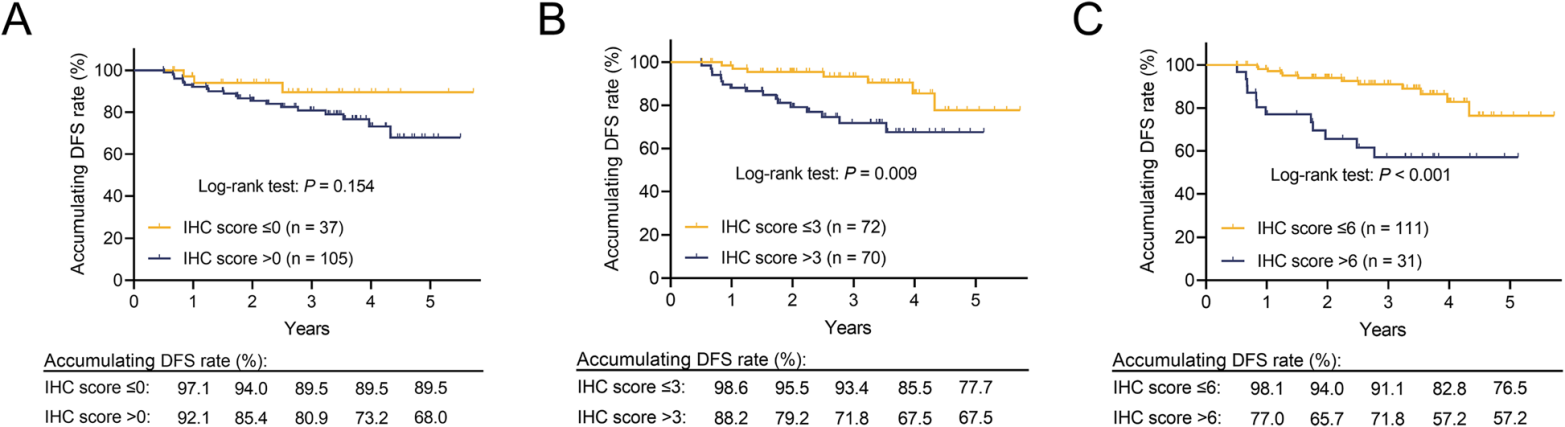
The ability of the tumor PLK4 IHC score to predict DFS in EC patients who underwent surgical resection. Comparison of DFS between EC patients who underwent surgical resection with tumor PLK4 IHC scores > 0 and ≤ 0 (**A**), those with tumor PLK4 IHC scores > 3 and ≤ 3 (**B**), and those with tumor PLK4 IHC scores > 6 and ≤ 6 (**C**)

In EC patients who underwent surgical resection, DFS did not vary between patients with tumor PLK4 IHC scores > 0 and ≤ 0 (*P* = 0.154, Fig. 2A). DFS was shortened in patients with a tumor PLK4 IHC score > 3 compared to those with a tumor PLK4 IHC score ≤ 3 (*P* = 0.009, Fig. 2B). Patients with a tumor PLK4 IHC score > 6 had worse DFS than those with a tumor PLK4 IHC score ≤ 6 (*P* < 0.001, Fig. 2C).

**Fig. 3:**
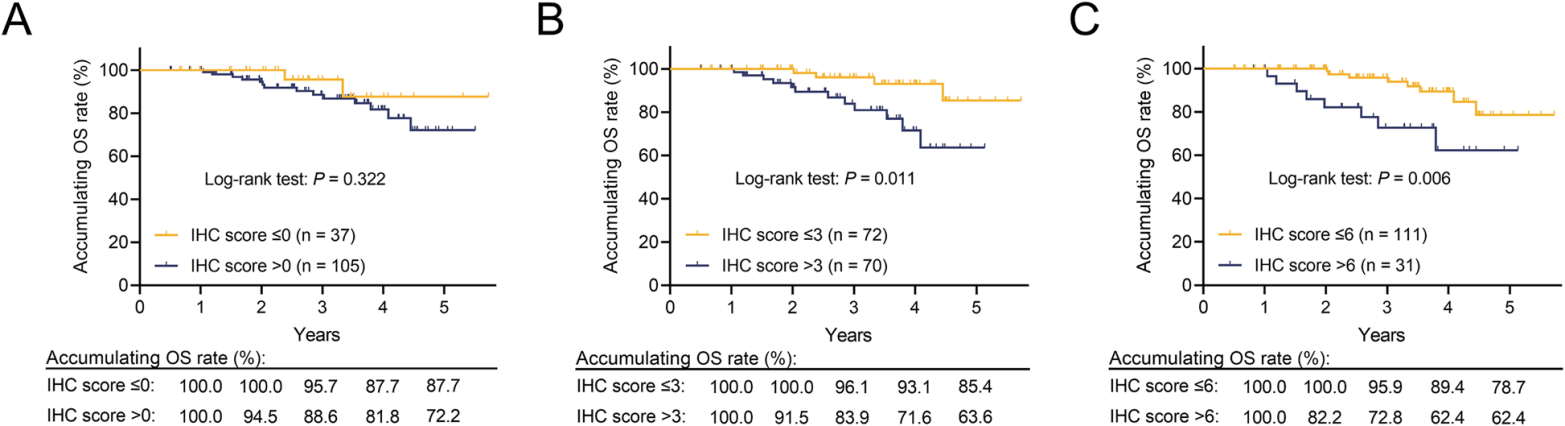
The ability of the tumor PLK4 IHC score to predict OS in EC patients who underwent surgical resection. Comparison of OS between EC patients who underwent surgical resection with tumor PLK4 IHC scores > 0 and ≤ 0 (**A**), those with tumor PLK4 IHC scores > 3 and ≤ 3 (**B**), and those with tumor PLK4 IHC scores > 6 and ≤ 6 (**C**)

There was no difference in OS between patients with PLK4 IHC scores > 0 and ≤ 0 (*P* = 0.322, Fig. 3A). OS was shortened in patients with PLK4 IHC scores > 3 compared to those with scores ≤ 3 (*P* = 0.011, Fig. 3B). Patients with a tumor PLK4 IHC score > 6 had worse OS than those with a score ≤ 6 (*P* = 0.006, Fig. 3C).

### Correlation of age, diabetes, and hypertension with DFS and OS

Age ≥ 60 years was not correlated with DFS (*P* = 0.077, Supplementary Fig. 1A), but it was associated with OS (*P* = 0.012, Supplementary Fig. 1B). Diabetes was not linked with DFS (*P* = 0.344, Supplementary Fig. 1C) or OS (*P* = 0.309, Supplementary Fig. 1D). Hypertension was not related to DFS (*P* = 0.200, Supplementary Fig. 1E) or OS (*P* = 0.135, Supplementary Fig. 1F) in EC patients who underwent surgical resection.

### Factors associated with DFS

In EC patients who underwent surgical resection, tumor PLK4 IHC score > 3 (vs. ≤ 3) (*P* = 0.013), tumor PLK4 IHC score > 6 (vs. ≤ 6) (*P* = 0.001), endometrioid carcinoma G3 (vs. endometrioid carcinoma G1/G2) (*P* = 0.003), serous endometrial carcinoma (vs. endometrioid carcinoma G1/G2) (*P* = 0.004), clear cell endometrial carcinoma (vs. endometrioid carcinoma G1/G2) (*P* = 0.002), myometrial invasion ≥ 50% (*P* = 0.015), stromal cervical invasion (vs. none or epithelia) (*P* = 0.001), lymphovascular invasion (*P* = 0.031), higher FIGO stage (*P* = 0.002), and adjuvant therapy (*P* = 0.031) were related to worse DFS. After adjustment, tumor PLK4 IHC score > 6 (vs. ≤ 6) (hazard ratio (HR): 3.156, *P* = 0.008), endometrioid carcinoma G3 (vs. endometrioid carcinoma G1/G2) (HR: 7.617, *P* = 0.002), serous endometrial carcinoma (vs. endometrioid carcinoma G1/G2) (HR: 5.393, *P* = 0.002), clear cell endometrial carcinoma (vs. endometrioid carcinoma G1/G2) (HR: 6.339, *P* = 0.003), and high FIGO stage (HR: 1.983, *P* < 0.001) were independently associated with shortened DFS (Table 3).

**Table 3:** Cox regression analysis of DFS

Features	*P* value	HR (95% CI)
**Univariable analysis**		
Tumor PLK4 IHC score		
>0 vs. ≤0	0.167	2.348 (0.700-7.882)
>3 vs. ≤3	0.013	3.060 (1.266-7.395)
>6 vs. ≤6	0.001	4.107 (1.843-9.153)
Age, ≥60 years vs. <60 years	0.084	2.086 (0.907-4.800)
Menopausal status, post-menopause vs. pre-menopause	0.169	2.340 (0.696-7.865)
Diabetes, yes vs. no	0.347	1.526 (0.632-3.683)
Hypertension, yes vs. no	0.205	1.688 (0.752-3.790)
Histological subtype		
Endometrioid carcinoma G1/G2 (reference)	1.000	
Endometrioid carcinoma G3 vs. endometrioid carcinoma G1/G2	0.003	5.415 (1.798-16.308)
Serous endometrial carcinoma vs. endometrioid carcinoma G1/G2	0.004	4.708 (1.656-13.386)
Clear cell endometrial carcinoma vs. endometrioid carcinoma G1/G2	0.002	6.714 (2.019-22.331)
Myometrial invasion ≥50%, yes vs. no	0.015	2.796 (1.223-6.393)
Cervical invasion, stromal vs. none or epithelia	0.001	3.741 (1.674-8.358)
Lymphovascular invasion, yes vs. no	0.031	2.427 (1.086-5.424)
Higher FIGO stage	0.002	1.729 (1.231-2.427)
CA125 abnormity, yes vs. no	0.178	1.740 (0.778-3.894)
CA19-9 abnormity, yes vs. no	0.315	1.533 (0.667-3.524)
CEA abnormity, yes vs. no	0.328	1.491 (0.670-3.322)
Adjuvant radiotherapy, yes vs. no	0.479	1.429 (0.531-3.843)
Adjuvant chemotherapy, yes vs. no	0.031	2.426 (1.086-5.424)
**Multivariable analysis**		
Tumor PLK4 IHC score, >6 vs. ≤6	0.008	3.156 (1.359-7.332)
Histological subtype		
Endometrioid carcinoma G1/G2 (reference)	1.000	
Endometrioid carcinoma G3 vs. endometrioid carcinoma G1/G2	0.001	7.617 (2.288-25.353)
Serous endometrial carcinoma vs. endometrioid carcinoma G1/G2	0.002	5.393 (1.877-15.500)
Clear cell endometrial carcinoma vs. endometrioid carcinoma G1/G2	0.003	6.339 (1.846-21.763)
Higher FIGO stage	<0.001	1.983 (1.361-2.889)

CA125 ≥35 U/mL, CA19-9 ≥37 U/mL and CEA ≥5 ng/mL was defined as abnormality

*DFS* disease-free survival, *HR* hazards ratio, *CI* confidence interval, *PLK4* polo-like kinase 4, *IHC* immunohistochemistry, *FIGO* the International Federation of Gynecology and Obstetrics, *CA125* cancer antigen 125, *CA19-9* cancer antigen 19-9, *CEA* carcinoembryonic antigen

### Factors associated with OS

In EC patients who underwent surgical resection, tumor PLK4 IHC score > 3 (vs. ≤ 3) (*P* = 0.018), tumor PLK4 IHC score > 6 (vs. ≤ 6) (*P* = 0.010), age ≥ 60 years (vs. < 60 years) (*P* = 0.019), endometrioid carcinoma G3 (vs. endometrioid carcinoma G1/G2) (*P* = 0.001), clear cell endometrial carcinoma (vs. endometrioid carcinoma G1/G2) (*P* < 0.001), stromal cervical invasion (vs. none or epithelia) (*P* = 0.014), lymphovascular invasion (*P* = 0.024), and high FIGO stage (*P* = 0.005) were linked with worse OS. After adjustment, tumor PLK4 IHC score > 3 (vs. ≤ 3) (HR: 3.918, *P* = 0.026), hypertension (HR: 3.108, *P* = 0.047), endometrioid carcinoma G3 (vs. endometrioid carcinoma G1/G2) (HR: 19.661, *P* < 0.001), clear cell endometrial carcinoma (vs. endometrioid carcinoma G1/G2) (HR: 30.569, *P* < 0.001), and high FIGO stage (HR: 2.413, *P* = 0.001) were independently associated with shortened OS (Table 4).

**Table 4:** Cox regression analysis of OS

Features	*P* value	HR (95% CI)
**Univariable analysis**		
Tumor PLK4 IHC score		
>0 vs. ≤0	0.333	2.082 (0.472-9.194)
>3 vs. ≤3	0.018	3.924 (1.259-12.228)
>6 vs. ≤6	0.010	3.619 (1.357-9.650)
Age, ≥60 years vs. <60 years	0.019	3.891 (1.249-12.120)
Menopausal status, post-menopause vs. pre-menopause	0.246	2.407 (0.546-10.600)
Diabetes, yes vs. no	0.315	1.722 (0.597-4.964)
Hypertension, yes vs. no	0.144	2.082 (0.779-5.566)
Histological subtype		
Endometrioid carcinoma G1/G2 (reference)	1.000	
Endometrioid carcinoma G3 vs. endometrioid carcinoma G1/G2	0.001	8.753 (2.325-32.952)
Serous endometrial carcinoma vs. endometrioid carcinoma G1/G2	0.082	3.585 (0.850-15.117)
Clear cell endometrial carcinoma vs. endometrioid carcinoma G1/G2	<0.001	17.499 (4.372-70.042)
Myometrial invasion ≥50%, yes vs. no	0.092	2.389 (0.867-6.586)
Cervical invasion, stromal vs. none or epithelia	0.014	3.452 (1.281-9.303)
Lymphovascular invasion, yes vs. no	0.024	3.136 (1.166-8.438)
Higher FIGO stage	0.005	1.822 (1.200-2.765)
CA125 abnormity, yes vs. no	0.530	1.374 (0.509-3.708)
CA19-9 abnormity, yes vs. no	0.756	1.185 (0.407-3.448)
CEA abnormity, yes vs. no	0.450	1.461 (0.547-3.904)
Adjuvant radiotherapy, yes vs. no	0.165	2.868 (0.649-12.671)
Adjuvant chemotherapy, yes vs. no	0.101	2.293 (0.852-6.171)
**Multivariable analysis**		
Tumor PLK4 IHC score, >3 vs. ≤3	0.026	3.918 (1.180-13.010)
Hypertension, yes vs. no	0.047	3.108 (1.013-9.529)
Histological subtype		
Endometrioid carcinoma G1/G2 (reference)	1.000	
Endometrioid carcinoma G3 vs. endometrioid carcinoma G1/G2	<0.001	19.661 (4.213-91.746)
Serous endometrial carcinoma vs. endometrioid carcinoma G1/G2	0.792	1.241 (0.248-6.207)
Clear cell endometrial carcinoma vs. endometrioid carcinoma G1/G2	<0.001	30.569 (6.359-146.950)
Higher FIGO stage	0.001	2.413 (1.438-4.049)

CA125 ≥35 U/mL, CA19-9 ≥37 U/mL and CEA ≥5 ng/mL was defined as abnormality

*OS* overall survival, *HR* hazards ratio, *CI* confidence interval, *PLK4* polo-like kinase 4, *IHC* immunohistochemistry, *FIGO* the International Federation of Gynecology and Obstetrics, *CA125* cancer antigen 125, *CA19-9* cancer antigen 19-9, *CEA* carcinoembryonic antigen

## Discussion

PLK4 plays a crucial role in cell division by facilitating centrosome duplication, affecting cytokinesis, responding to DNA damage, and maintaining genome stability [16]. Moreover, it contributes to ciliogenesis and metastasis, fostering cancer development [9]. PLK4 is associated with tumor characteristics [17–19]. For example, one study showed that increased PLK4 mRNA expression is linked with tumor stage in non-small cell lung cancer patients [17]. Another study indicated that increased PLK4 mRNA expression is associated with tumor size ≥ 7 cm and high tumor node metastasis (TNM) stage in renal cell carcinoma [18]. A study elucidated that PLK4 mRNA expression is linked with a higher incidence of lymph node metastasis and distant metastasis or surrounding recurrence in breast cancer [19].

This study revealed that tumor PLK4 protein expression was positively associated with lymphovascular invasion and FIGO stage in EC patients who underwent surgical resection. The possible explanations could be as follows: To begin with, PLK4 might promote EC cell invasion and metastasis by regulating actin-related protein 2/3-mediated actin cytoskeletal rearrangement [20]. Besides, PLK4 might induce epithelial-mesenchymal transition (EMT) by regulating the Wnt/β‑catenin signaling pathway, enhancing tumor invasion and metastasis [21]. Therefore, elevated tumor PLK4 protein expression is related to lymphovascular invasion in EC patients who underwent surgical resection. In addition, PLK4 facilitates centrosome duplication, accelerates cytoskeletal rearrangement and EMT, and contributes to tumor cell proliferation, invasion, and metastasis [16, 20, 21]. Therefore, increased tumor PLK4 protein expression is linked with elevated FIGO stage in EC patients who underwent surgical resection.

The negative linkage of PLK4 with survival in many cancers has been reported [22–24]. Taking one study as an example, high levels of PLK4 transcripts are linked with poor relapse-free survival in patients with breast cancer [22]. Another study indicated that high PLK4 expression is associated with unfavorable survival in glioma patients [23]. High expression of PLK4 is an independent factor predicting poor OS in patients with colorectal cancer [24]. Consequently, PLK4 is also speculated to be a prognostic biomarker in EC patients.

This study revealed that elevated tumor PLK4 IHC scores (> 3 and > 6) were associated with shortened DFS and OS in EC patients who underwent surgical resection, which could be explained as follows: First, PLK4 promotes cancer cell migration and metastasis, resulting in aggravated progression of EC and shortened DFS [25]. Second, PLK4 is related to lymphovascular invasion and higher FIGO stage, which could lead to worse OS. Taken together, these results indicate that tumor PLK4 protein expression is negatively linked with survival in EC patients who underwent surgical resection. Further multivariable Cox regression analyses suggested that high tumor PLK4 protein expression (> 6 and > 3) is independently predicts worse DFS and OS, respectively, which provided evidence for the subsequent clinical application of PLK4 in EC patients who underwent surgical resection, but its optimal cutoff value needs more investigation. Moreover, this study also found that aggressive tumor subtypes (endometrioid carcinoma G3, serous endometrial carcinoma, clear cell endometrial carcinoma) (vs. endometrioid carcinoma G1/G2), hypertension, and high FIGO stage were independently associated with shortened DFS or OS in EC patients who underwent surgical resection, which was consistent with the results of previous studies [26–29].

Interestingly, artificial intelligence, especially radiomic analysis of radiological images, represents a current topical issue in EC investigation [30]. The integration of radiomic analysis with other clinical diagnostic measures holds the potential to enhance preoperative risk stratification, diagnosis, treatment, and prognosis of EC [30]. Consequently, the clinical value to combine PLK4 with artificial intelligence in the management of EC patients warrants future investigation. Moreover, molecular/genomic analysis is another valuable method for tailoring treatments of EC [31]. According to molecular characterization, EC could be divided into four distinct groups (including polymerase epsilon ultramutated, microsatellite instability hypermutated, copy-number low, and copy-number high), each exhibiting different prognosis in terms of progression and recurrence risk [32]. Therefore, integrating PLK4 with molecular analysis is another potential direction for our future investigations.

Furthermore, considering older age and comorbidities as risk factors for EC prognosis [33], this study analyzed the correlation of age, diabetes, and hypertension with the survival and revealed that age ≥ 60 years was associated with shortened OS in EC patients who underwent surgical resection. The possible explanation could be that older patients (aged ≥ 60 years) often have reduced physical condition, which affects their rehabilitation after surgical resection and subsequently influences their survival [34]. On the other hand, this study also showed that diabetes and hypertension were not linked with DFS or OS in EC patients who underwent surgical resection. The probable reason could be that diabetes and hypertension are common chronic diseases that potentially affect the survival of EC patients. While patients with these chronic diseases often receive corresponding pharmacotherapy to control their blood glucose levels and blood pressure. Thereby, diabetes and hypertension were not related to DFS or OS in EC patients who underwent surgical resection.

This study was the first study determining PLK4 expression in EC patients who underwent surgical resection and setting several cutoff values to analyze its prognostic value. However, some limitations still existed in the current study. First, the sample size of this study was relatively small, which could weaken the statistical power. Besides, this study was a retrospective, single-center study, leading to unavoidable selective bias. Thereby, multiple center studies with a larger sample size were warranted to validate the results. In addition, this study only enrolled EC patients who underwent surgical resection; thus, the findings were not applicable to those who opted for nonsurgical interventions to preserve fertility. Moreover, the screening for Lynch syndrome, a risk factor for EC occurrence [35], was not performed in the current study, and therefore, the association of PLK4 with Lynch syndrome in EC patients who underwent surgical resection could not be determined. Fifth, comorbidities might impact EC outcomes [33]. Nevertheless, this study only included data of diabetes and hypertension, thereby, the comprehensive influence of comorbidities on EC outcomes was unclear. Finally, the mechanism of PLK4 in EC remains unclear and requires further in vitro and in vivo study.

## Conclusion

In summary, tumor PLK4 protein expression reflects lymphovascular invasion (*P* = 0.008) and higher FIGO stage (*P* = 0.005) in EC patients who undergo surgical resection. Moreover, its IHC score > 6 (*P* = 0.008) or > 3 (*P* = 0.026) serves as an independent indicator for shortened DFS and OS in these patients, respectively, suggesting its ability for predicating disease recurrence and death of these patients. The findings of this study provide a potential clinical reference for personalized treatment decision in EC patients who undergo surgical resection. Prospectively, the integration of PLK4 with artificial intelligence and molecular analysis in EC management is a potential direction for future investigations.

### Supplementary Information

**Additional file 1:** **Supplementary Figure 1A-B:** Correlation of age with DFS and OS in EC patients who underwent surgical resection; **Supplementary Figure 1C-D:** Correlation of diabetes with DFS and OS in EC patients who underwent surgical resection; **Supplementary Figure 1E-F:** Correlation of hypertension with DFS and OS in EC patients who underwent surgical resection.

## Data Availability

The datasets used and/or analyzed during the current study are available from the corresponding author upon reasonable request.
