# Supplementary figures and images for: Tumor polo-like kinase 4 protein expression reflects lymphovascular invasion, higher Federation of Gynecology and Obstetrics stage, and shortened survival in endometrial cancer patients who undergo surgical resection
Source: BMC Womens Health. 2024 Feb 7;24:101. doi: 10.1186/s12905-024-02911-9 (PMC10851612; doi:10.1186/s12905-024-02911-9)

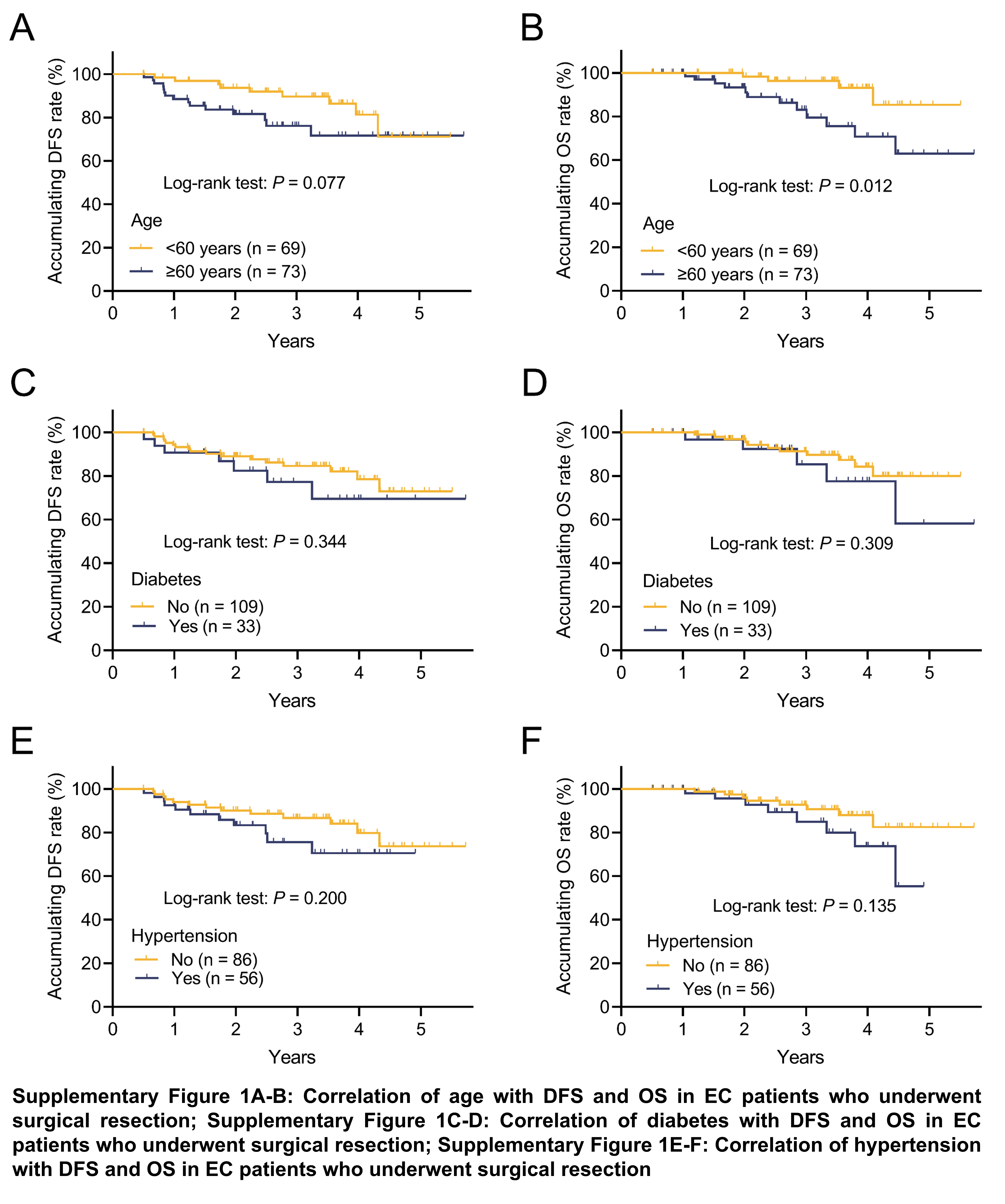

Supplement: Supplementary file 1 — Additional file 1: Supplementary Figure 1A-B: Correlation of age with DFS and OS in EC patients who underwent surgical resection; Supplementary Figure 1C-D: Correlation of diabetes with DFS and OS in EC patients who underwent surgical resection; Supplementary Figure 1E-F: Correlation of hypertension with DFS and OS in EC patients who underwent surgical resection. [file 12905_2024_2911_MOESM1_ESM.tif]
